# Using Assisted Partner Services for HIV Testing and the Treatment of Males and Their Female Sexual Partners: Protocol for an Implementation Science Study

**DOI:** 10.2196/27262

**Published:** 2021-05-20

**Authors:** Edward Kariithi, Monisha Sharma, Emily Kemunto, Harison Lagat, George Otieno, Beatrice M Wamuti, David A Katz, Christopher Obong'o, Paul Macharia, Rose Bosire, Sarah Masyuko, Mary Mugambi, Carol E Levin, Wenjia Liu, Unmesha Roy Paladhi, Bryan J Weiner, Carey Farquhar

**Affiliations:** 1 PATH-Kenya Kisumu Kenya; 2 Department of Global Health University of Washington Seattle, WA United States; 3 Kenya Medical Research Institute Nairobi Kenya; 4 Ministry of Health Nairobi Kenya; 5 School of Nursing University of Washington Seattle, WA United States; 6 Department of Epidemiology University of Washington Seattle, WA United States

**Keywords:** implementation science, assisted partner notification services, HIV testing and counseling, linkage to care, western Kenya

## Abstract

**Background:**

Despite the effective scale-up of HIV testing and treatment programs, only 75% of people living with HIV (PLWH) globally know their status, and this rate is lower among men. This highlights the importance of implementing HIV testing and linkage interventions with a high uptake in this population. In a cluster randomized controlled trial conducted in Kenya between 2013 and 2015, we found that assisted partner services (APS) for HIV-exposed partners of newly diagnosed PLWH safely reached more HIV-exposed individuals with HIV testing compared with client referral alone. However, more data are needed to evaluate APS implementation in a real-world setting.

**Objective:**

This study aims to evaluate the effectiveness, acceptability, fidelity, and cost of APS when integrated into existing HIV testing services (HTS) in Western Kenya.

**Methods:**

Our study team from the University of Washington and PATH is integrating APS into 31 health facilities in Western Kenya. We are enrolling females newly diagnosed with HIV (index clients) who consent to receiving APS, their male sexual partners, and female sexual partners of male sexual partners who tested HIV positive. Female index clients and sexual partners testing HIV positive will be followed up at 6 weeks, 6 months, and 12 months postenrollment to assess linkage to care, antiretroviral therapy initiation, and HIV viral load suppression. We will evaluate the acceptability, fidelity, and cost of real-world implementation of APS via in-depth interviews conducted with national, county, and subcounty-level policy makers responsible for HTS. Facility health staff providing HTS and APS, in addition to staff working with the study project team, will also be interviewed. We will also conduct direct observations of facility infrastructure and clinical procedures and extract data from the facilities and county and national databases.

**Results:**

As of March 2020, we have recruited 1724 female index clients, 3201 male partners, and 1585 female partners. We have completed study recruitment as well as 6-week (2936/2973, 98.75%), 6-month (1596/1641, 97.25%), and 12-month (725/797, 90.9%) follow-up visits. Preliminary analyses show that facilities scaling up APS identify approximately 12-18 new HIV-positive males for every 100 men contacted and tested. We are currently completing the remaining follow-up interviews and incorporating an HIV self-testing component into the study in response to the COVID-19 pandemic.

**Conclusions:**

The results will help bridge the gap between clinical research findings and real-world practice and provide guidance regarding optimal strategies for APS integration into routine HIV service delivery.

**International Registered Report Identifier (IRRID):**

DERR1-10.2196/27262

## Introduction

The HIV epidemic continues to cause significant morbidity and mortality, disproportionately affecting sub-Saharan Africa (SSA), where most HIV infections occur [1]. Approximately 65% of people living with HIV (PLWH) globally know their status, suggesting that expanding targeted testing strategies are needed to achieve the first 95 of the Joint United Nations Programme on HIV/AIDS ambitious testing, treatment, and viral suppression targets [2]. PLWH in SSA are largely diagnosed through facility-based HIV testing; however, testing coverage is lacking and insufficient to curb the HIV epidemic, particularly in men and vulnerable populations [3]. Barriers to facility testing include distance, costs, and confidentiality concerns, which result in many PLWH presenting late for care when they are already symptomatic or at advanced stages of the disease [4,5].

Assisted HIV partner services—providers contacting and testing sexual and injecting partners of people diagnosed with HIV—can be an efficient strategy for diagnosing people with HIV, linking them to HIV care and prevention [6,7]. In 2016, the World Health Organization recommended offering assisted partner services (APS) to all PLWH to close the gap in HIV testing coverage [8], and these services are now rapidly scaling up globally. Partner contacting and testing can be conducted through (1) client referral—newly diagnosed individuals (index clients) are asked to notify their partners of exposure and encourage HIV testing; (2) provider referral—providers contact partners and offer testing while ensuring the clients’ confidentiality; and (3) contract referral—index clients and providers agree on a set amount of time for clients to notify partners, after which providers contact partners and offer testing [9]. In practice, APS are implemented as a combination of these options.

Clinical trials and demonstration projects of APS in SSA have found high HIV positivity (30%-63%) among sexual partners of index clients and high median CD4 counts at diagnosis, indicating that individuals are identified earlier in their disease course compared with those identified by facility-based testing [3,10-13]. Early case detection and linkage to care can improve clinical outcomes [11,14,15]. Mathematical modeling analyses indicate that APS are a cost-effective strategy for reducing HIV burden in SSA [16]. In addition, APS are an effective method to reach men through their HIV-positive female partners, as women are tested for HIV at higher rates than men [17]. Men in SSA are more likely to start antiretroviral therapy (ART) at advanced disease stages and consequently have poorer clinical outcomes compared with women [18,19]. Low male testing and treatment rates also serve to increase HIV transmission to their female partners.

Despite its demonstrated efficacy, scaling up APS presents challenges. The translation of findings from randomized controlled trials into real-world settings can be a challenge because of weaknesses in health system structures and differences in intervention delivery, monitoring, and available resources [20]. The implementation of science evaluations of APS offers a real-world approach using existing systems to scale up interventions. As a critical next step in bringing APS to scale in Kenya and across SSA, this study uses implementation science methods to assess the effectiveness and feasibility of APS and generates evidence to inform rapid and sustainable implementation across the region.

Our objective is to evaluate the effectiveness, feasibility, and cost of implementing APS integrated within routine HIV testing services (HTS) in Western Kenya, a region with high HIV prevalence (>15%) [21]. Specifically, we aim to (1) evaluate the effectiveness of integrated APS and (2) determine the integration, implementation fidelity, acceptability, demand, and costs of implementing APS. By identifying health facility and individual-level factors that influence the uptake of HIV testing, linkage to care, and fidelity to APS, we will evaluate how these factors inform the successful scale-up of APS in Kenya.

## Methods

### Study Design

This hybrid type 2 implementation science study [22] has two aims: (1) to determine the effectiveness of APS when integrated within routine HTS and (2) to evaluate the implementation of APS in these settings, including the integration, implementation fidelity, acceptability, demand, and costs of the intervention. The study leveraged existing integrated HIV prevention, care, and treatment platforms from the Afya Ziwani project, the existing President’s Emergency Plan for AIDS Relief–funded and PATH. PATH is a local nongovernmental organization in Western Kenya. HTSs are provided in facility and community settings, including *safe spaces* where DREAMS (Determined, Resilient, Empowered, AIDS-free, Mentored, and Safe) interventions are provided to vulnerable adolescent girls and young women.

The DREAMS program aims to reduce new HIV cases by providing girls and young women at the highest risk of HIV infection and their partners, families, and communities with a tailored, comprehensive, and evidence-informed HIV service package, including HTS. Afya Ziwani provides technical implementation support to health facilities that serve as sites for study enrollment. Nine safe spaces have been created within the catchment areas of health facilities that provide a variety of services, including HTS, by staff for adolescent girls and young women. Excluding the antenatal care clinics, around 50-80 persons test HIV positive each month in larger facilities and 10-20 in smaller facilities, with more than 50% of newly diagnosed individuals being female. In 2018, HIV prevalence in Homa Bay and Kisumu counties was estimated at 19.6% and 17.5%, respectively, in the general population [23]. Therefore, this is a high number of girls and women with HIV who are enrolled in health facilities and linked safe spaces. This study integrated APS into the existing infrastructure of Afya Ziwani in collaboration with county and subcounty health management teams in Homa Bay and Kisumu counties.

The study was approved by the ethics and research committee of Kenyatta National Hospital (P465/052017) and the University of Washington institutional review board (STUDY00002420). No payment was provided to the participants for their participation. Breaches in confidentiality, study protocol, or adverse events attributable to this study were reported to both ethics and research committees and institutional review boards. The findings of this study will be disseminated to the Kenya Ministry of Health (MOH) and Kenya National AIDS and Sexually Transmitted Infection Control Programme (NASCOP) through direct communication and technical working group meetings and to the greater scientific and public health community through national and international conferences and peer-reviewed manuscripts in academic journals.

### Aim 1: To Evaluate the Effectiveness of Integrated APS

#### Study Sites

Table 1 shows the distribution of study sites where HTS was provided and study activities were conducted. These facilities were in four Homa Bay County and five Kisumu County wards, encompassing a total of 31 study sites, some of which were connected to DREAMS safe spaces and nine additional safe spaces supported by the Afya Ziwani project.

**Table 1:** Distribution of 31 study sites in Western Kenya.

Facility name	Private or public	Subcounty	County
Kabondo Sub-County Hospital	Public	Kabondo	Homa Bay
Kauma Health Centre	Public	Kabondo	Homa Bay
Nyangiela Sub-County Hospital	Public	Kasipul	Homa Bay
Othoro Sub-County Hospital	Public	Kabondo	Homa Bay
Rachuonyo County Hospital	Public	Kasipul	Homa Bay
Tala Dispensary	Public	Kabondo	Homa Bay
Atela Dispensary	Public	Kabondo	Homa Bay
Kasewe Dispensary	Public	Kabondo	Homa Bay
Kokwanyo Health Centre	Public	Kabondo	Homa Bay
Matata Hospital	Private	Kasipul	Homa Bay
Nyalgosi Dispensary	Public	Kasipul	Homa Bay
Ober Sub-County Hospital	Public	Kabondo	Homa Bay
Ombek Dispensary	Public	Kasipul	Homa Bay
Nyawango Health Centre	Public	Kasipul	Homa Bay
Kimonge Dispensary	Public	Kabondo	Homa Bay
Airport Health Centre	Public	Kisumu West	Kisumu
Kowino Dispensary	Public	Kisumu East	Kisumu
Ojola Health Centre	Public	Kisumu West	Kisumu
Port Florence Hospital	Private	Kisumu West	Kisumu
Simba Opepo Dispensary	Public	Kisumu East	Kisumu
St. Elizabeth Chiga Hospital	Private	Kisumu West	Kisumu
Chiga Dispensary	Public	Kisumu West	Kisumu
Gita Sub-County	Public	Kisumu West	Kisumu
Migosi Sub-County Hospital	Public	Kisumu Central	Kisumu
Nyalenda Health Centre	Public	Kisumu Central	Kisumu
Ober Kamoth Sub-County Hospital	Public	Kisumu West	Kisumu
St. Mark’s Lela Sub-County Hospital	Public	Kisumu West	Kisumu
Ring Road Dispensary	Private	Kisumu East	Kisumu
Disciples of Mercy	Mission	Kisumu East	Kisumu
Usoma Dispensary	Public	Kisumu West	Kisumu
Nightingale Hospital	Private	Kisumu Central	Kisumu

#### Study Participants

We planned to enroll up to 8000 female index participants (girls and women who tested positive for HIV at the facilities) and 10,000 sexual partners across study sites. The target enrollment was 2000 in year 1 of the study and 3000 per year in years 2 and 3. The inclusion criteria for the index participants were (1) female, (2) testing HIV positive and not in care or on treatment, (3) aged ≥18 years or emancipated minor (girls aged ≥15 years who are married; pregnant; or have had a sexually transmitted infection, including HIV), (4) willing to participate in the study, and (5) willing to provide contact information of ≥1 sex partner. Pregnant women, those younger than 15 years, and individuals who reported intimate partner violence (IPV) within the past month of enrollment were excluded. History of IPV was determined using an IPV screening questionnaire that included questions about emotional, physical, and sexual violence (Multimedia Appendix 1). Male sexual partners of index clients who tested HIV positive also received APS and their other female partners were notified of their potential HIV exposure and offered HTS.

#### Study Procedures

##### Index Participant Recruitment and Enrollment

Recruitment of index participants occurred at the study sites. Females testing HIV positive at the participating sites were provided information on the study, including an overview of APS and study procedures in English or local languages (Swahili and Luo) by HTS providers. The HTS staff screened the interested participants for eligibility and were asked to provide written informed consent if eligible. Enrolled participants were interviewed using structured forms regarding their demographic characteristics, sexual behavior, substance use, HIV testing history, and the number of male sex partners in the past 3 years. HTS providers collected contact information for all male sex partners in the past 3 years and emphasized that all information would be kept confidential and that the index cases’ identities would not be revealed when contacting their partners.

##### APS Procedures

HTS providers called all elicited male sexual partners to inform them of their HIV exposure and offer HIV testing at the facility or a convenient location in the community. Initially, up to three attempts were made to notify the partners by phone; if unsuccessful, or if the female index client did not provide a phone number for the partner, HTS providers physically traced the partners in the community. If unsuccessful, two further attempts were made to notify the partners, either by phone or in-person. Partners were classified as lost to follow-up after six unsuccessful attempts or if a partner refused to meet with an HTS provider.

When implementing APS, HTS providers informed the partners of their potential HIV exposure without identifying the index participant. They asked the partners about their HIV testing history, provided HIV counseling, and offered HIV testing. Partners with a prior HIV diagnosis were asked about their current HIV care, and those who were out of care were counseled about the benefits and locations to access care. If a notification occurred over the phone, partners were encouraged to seek HTS at a study site or offered the opportunity for study staff to conduct a home-based HIV test. For partners who chose to seek HTS at a facility or safe space, the HTS provider who conducted the initial outreach generally contacted the partners via telephone to remind them of their testing appointment, rescheduled appointments as needed, conducted HTS, and documented test results, even if HTS was conducted at nonstudy sites. If HTS was completed at a nonstudy site by the partner, results were obtained via self-report. If a notification occurred as part of a home visit, partners were offered HTS after verbal consent per Kenya national guidelines [24]. When possible, HTS providers accompanied HIV-positive partners to the nearest HIV comprehensive care center for linkage to care.

##### Partner Participant Recruitment and Enrollment

Partners were aged ≥18 years. Recruitment and enrollment of partner participants took place during the tracing visit. Before testing for HIV, partners were invited to enroll in the study; however, they did not need to enroll to get tested. When possible, male partners who tested HIV positive were offered APS for their other female sexual partners.

##### Participant Follow-Up

HTS providers made phone calls to assess linkage to and engagement in care and ART initiation for index clients and partners with HIV at 6 weeks and 6 months postenrollment. At 12 months postenrollment, participants were asked to make a physical visit to the comprehensive care center for clinical care consultation and to conduct an HIV viral load test. For all follow-up timepoints, participants who were not reached after three attempts by phone were physically traced.

##### IPV Monitoring

Adolescent girls and women reporting IPV within the past month were excluded from participation and instead referred to IPV counseling and other support services, as needed. Those reporting that they had not experienced recent IPV but feared IPV and abuse from a partner were eligible for study participation but had the option to refuse both APS and study participation. They received instructions regarding confidential reporting to clinical providers and study staff, and case report forms documenting any IPV were completed by phone or in person, depending on their preference. We provided additional training on IPV counseling for study staff and ensured that resources had been identified at all sites to safely refer females who reported abuse or concern for their safety. Individuals experiencing IPV were excluded at enrollment to avoid any harm as a result of notifying their partners. However, if they reported IPV at a follow-up visit (after enrollment), they were followed to ensure that they were safe and referred to IPV services as needed. In addition, these data will be helpful in studying the safety of APS.

#### Outcomes

The effectiveness outcomes included (1) number of persons testing for HIV for the first time among all tested, (2) male partners who are newly diagnosed with HIV versus known to be living with HIV among all tested, (3) linkage to care and initiation of ART by 6 weeks and follow-up at 6 months for male partners with HIV and female index clients, and (4) suppression of plasma HIV RNA levels to <400 copies/µL at 12 months among male partners living with HIV and female index clients. We also monitor adverse events, including IPV and relationship dissolution.

#### Data Collection

Study data were collected using the open-source Open Data Kit platform using questionnaires administered on Android smartphones or tablets [25]. Data were encrypted for storage on the devices and transferred immediately over an encrypted connection to a secure server at NASCOP. The study database was backed up nightly to a secure server at the University of Washington.

#### Data Analysis

We will use log-binomial regression with robust standard errors to assess the proportion of participants with the main outcomes of interest and use multivariate log-binomial and logistic regression to examine the potential predictors of outcomes of interest, including participant demographics, HIV testing history, sexual behavior, and location. We will conduct time trend analyses to examine the changes in proportions with outcomes of interest over time as the APS are scaled up.

### Aim 2: To Determine the Fidelity, Integration, Acceptability, Demand, Technical Efficiency, and Cost of Implementing APS

#### Study Sites

The aim 2 implementation evaluation occurred in (1) Homa Bay and Kisumu HTS facilities and DREAMS sites; (2) selected subcounty and county administrative offices linked to health facilities; and (3) NASCOP’s national administrative offices in Nairobi, Kenya. We included both high-performing and low-performing facilities and covered the range of facility levels from small rural outposts to high-volume urban clinics.

#### Study Participants

HTS providers involved in tracing and HIV testing of partners underwent in-depth interviews (IDIs) with a qualified qualitative interviewer for the evaluation of acceptability of APS, perceived community demand for APS, and implementation fidelity to the APS protocol. The enrolled index participants and male partners who had received APS also underwent IDIs to evaluate the acceptability and perceived demand for APS. Key APS stakeholders, including facility in-charges, county or subcounty AIDS/sexually transmitted infection coordinators, and administrators at NASCOP, were interviewed to evaluate the integration and perceived demand for APS.

#### Study Procedures

HTS providers, index and partner participants, and key APS stakeholders were informed of and invited to participate in the study. Those interested were screened for eligibility and provided consent for an IDI. IDIs were conducted in the language preferred by an experienced qualitative researcher who spoke English, Swahili, and the local languages in a quiet place at a study facility or another location chosen by the participant.

#### Outcomes

Key outcomes for aim 2 included the determination of (1) health facility and individual-level factors that influence fidelity to APS; (2) acceptability, demand, and health system requirements influencing the feasibility of APS; and (3) costs of APS when integrated into existing HTS. Cost metrics included total incremental economic costs of APS, cost per person traced, cost per person HIV tested, and cost per person linked to ART.

#### Data Collection

##### Implementation Fidelity

Although we anticipated some variation in APS implementation across sites, it was difficult to know how contextual differences in the execution of APS procedures would affect outcomes unless we examined how well the implemented intervention matched the intended implementation. We used a conceptual framework for implementation fidelity to identify and describe the key implementation fidelity elements [26].

A convergent mixed methods approach was used to concurrently collect qualitative and quantitative data. A total of two data sources, facility data and staff, were used to triangulate the implementation fidelity to the APS. Facility-level data were collected daily by HTS providers to ascertain the frequency, type, and success of each tracing attempt using structured questionnaires. Measurement of fidelity started at least 12 months after site activation to provide enough time for HTS providers to familiarize themselves with the APS intervention. In addition, as a staggered study started in the 2 counties, with Homa Bay sites initiating 6 months after Kisumu sites, the 12-month period ensured that HTS providers had significant exposure to APS in both counties before assessment.

##### Acceptability of and Demand for APS

We examined APS acceptability among HTS providers, index clients, and male partner participants. We conducted 14 IDIs with HTS providers (same population in the implementation fidelity aim) and 32 IDIs with clients (16 index clients and 16 partners) to address questions related to APS acceptability from 8 purposively selected facilities that vary in patient volume and APS performance. We selected 1 female index who elicited ≤2 male partners and another female index who elicited >2 male partners at each facility in both Homa Bay and Kisumu. Interview guides addressed APS satisfaction, perceived benefits of the intervention, and challenges that may affect delivery or uptake.

To assess demand, we conducted IDIs with 14 HTS providers, 32 clients, and 20 key stakeholders (same population in the integration aim) to assess experiences with provision, administrative oversight, and use of APS intervention activities. Guiding questions were “To what extent is APS likely to be used or supported, and how much demand for APS is perceived to be there in people (general and specific groups) in the community?” In addition, APS delivery statistics provided information on the actual usage of the services.

All IDIs were conducted by a qualified qualitative interviewer using a semistructured interview guide and audio recorded and transcribed for analysis.

##### Integration

We adapted the analytic framework by Grepin and Reich to develop and assess a strategy for integrating APS into existing HIV programs, [27] measure the extent of integration. Integration was measured by the extent of coordination, collaboration, and consolidation, occurring at various levels (policy, organizational, national or regional, and local). We conducted 20 IDIs with purposively selected key NASCOP policy makers, subcounty and county health management team members, facility staff, and community representatives at study initiation and 1 year into APS scale-up to identify opportunities and challenges for both integration within Kenya’s current HIV cascade of care and extent of integration over time. The findings were used to develop metrics to monitor the extent to which integration occurs at each level during APS implementation, along with specific scoring criteria.

##### Cost

We conducted a microcosting and time-in-motion observation of APS activities. Start-up and recurrent cost data were obtained from financial records, including budgets and expense reports from the MOH, PATH-Kenya, and study sites. We collected information on the quantities of inputs and prices of each input used to implement enhanced APS services, including personnel, commodities, and capital goods (such as vehicles, computers, phones, and other equipment). We also obtained budget expense report data on program design, adaptation, and installation related to awareness raising, materials development, training, and the costs of other start-up activities that are essential for expanding the program. The team sought to obtain information directly from the study sites whenever possible. However, if this information was not available, estimates were used from the MOH records of facilities with similar characteristics. Cost data were synthesized using Microsoft Excel spreadsheets.

##### Technical Efficiency of Health System Requirements

Technical efficiency is a relative measure that compares the inputs used (human, technological, and financial) with the outputs attained (number and level of services) [28]. It is designed to assess whether an organization is deploying the right mix of personnel, equipment, supplies, and facilities to produce outputs at the lowest cost. Using data collected from the cost aim, we examined the necessary health system requirements needed to operate APS provision, identify environmental constraints, and determine their technical efficiency. The domains examined were (1) facility-level characteristics (organizational structure, management, governance, decision-making processes, funding sources, training, supervision, incentives, and accountability) and (2) environmental contextual constraints (size of target population and population actually using the services). Determinants of efficiency are those that affect the cost of delivery, which depends on a facility’s performance.

#### Data Analysis

##### Implementation Fidelity

Using a convergent mixed methods approach, we will examine the health facility characteristics (eg, location or staffing) associated with high fidelity to protocol elements and positive implementation outcomes by triangulating facility-level data with qualitative findings from the HTS provider IDIs. IDI audio recordings will be transcribed and analyzed by 2 independent coders using thematic content analysis to determine the key implementation fidelity themes using both deductive and inductive coding [29].

Descriptive statistics will be used to describe the characteristics of participants by county, type, and success of tracing attempts. Categorical variables will be described using counts and proportions, and continuous variables will be described using medians and IQRs. The time needed to conduct tracing attempts will be described using median and IQR and compared with the standard APS protocol to determine the fidelity to the protocol. Pareto charts based on the Pareto principle that 80% of the effects originate from 20% of the causes will be used to determine the tracing attempts through which most clients are successfully traced [30]. Data collected from HTS providers will be used to describe contextual factors.

In the multivariate analysis, successful tracing attempts (coded as either yes or no) will be the outcome of interest. We will consider two levels of data: individual level with clustering at the facility level. Log-binomial generalized estimating equations with exchangeable correlation structure and robust SEs will be used to estimate the relative risks and 95% CIs of successful attempts by the type of tracing attempt (phone vs physical). Variables associated with linking to care in these univariate analyses (*P*<.10) will be included simultaneously in the multivariate model. Moderators will not be included in this multivariate model; however, they will be used to provide context to the results of the quantitative analysis.

##### Acceptability and Demand of APS

Recordings of the interviews will be transcribed verbatim and translated into English. Codebooks will be developed and tested using the first five to six interview transcripts and applied to all transcripts for coding, once finalized. Transcripts will be independently coded by two analysts who will reach consensus through discussion. Disagreements will be adjudicated by a third qualitative researcher. Qualitative data will be analyzed thematically using an inductive coding approach [29]. Analysis will be conducted first for all interviews and then we will perform a comparative analysis among different participant groups and different types of facilities varying in patient volume and APS performance.

##### Cost

Microcosting data will be used to estimate total incremental costs by facility and all-sample-weighted average incremental cost per partner traced, cost per partner tested, and cost per partner newly diagnosed as HIV positive. We will estimate the total incremental and unit costs for each facility and estimate the average and weighted cost metrics for the facilities in our sample. We will generate cost profiles based on activity and input. Using a government payer perspective, we will construct an Excel-based static deterministic model to simulate the budget impact analysis of APS on an annual basis over a 5-year time horizon using HIV prevalence estimates from Kisumu and Homa Bay counties. We will assume that 70% of this population will test for HIV and that 50% of these will receive APS.

Cost estimates for budget impact analysis, will include incremental costs for APS, ART, clinic visits, and hospitalization. We will compare two scenarios, assuming 50% and 100% APS implementation within the health facilities. We will assume that HTS providers working a 5-day work week will have a case load of no more than 10 clients per day per counselor based on national Kenyan HTS standards and previous studies.

### Study Registration

The study was registered on clinicaltrials.gov on June 22, 2017. Enrollment started in May 2018 in Kisumu County and in November 2018 in Homa Bay County. The study is expected to be completed in May 2022.

## Results

As of March 2020, we recruited 1724 index clients, 3201 male partners, and 1585 female partners. We completed recruitment for this study and 6-week (2936/2973, 98.75%), 6-month (1596/1641, 97.25%), and 12-month (725/797, 90.9%) follow-up visits. We are now completing the remaining 12-month follow-up visits. Preliminary analyses demonstrate that by scaling up APS, facilities can identify 12-18 new HIV-positive males for every 100 men contacted and tested. This is considerably higher than the average HTS yield in Kenya of <2%. During this period, 21 individuals were ineligible for the APS study because of IPV concerns. Despite this, there have been 35 IPV events related to HTS and APS, which were not due to procedures. Overall, 32 relationships were dissolved during this period.

## Discussion

The Kenya MOH and NASCOP guidelines for HIV testing recommends voluntary APS implementation as part of routine HTS. This study is designed to assess how best to implement and scale up APS within public health settings in low-income countries such as Kenya. We have integrated study activities and follow-up visits into existing routine HIV testing, prevention, and care programs at participating sites to assess how APS functions at scale in a real-world setting.

With the current global goal of reaching 95-95-95 in HIV epidemic control, the pool of undiagnosed PLWH continues to decline, and APS are an effective modality for reaching those who are undiagnosed. This is especially true for certain subgroups, such as men, who are more likely to be missed by standard HTS. In addition, the study documents other aspects of APS implementation, such as cost, fidelity, and acceptability, which will inform a costed national scale-up of this modality. We sought to identify the most critical factors for successful APS implementation by rigorously documenting process outcomes such as provider elicitation rates and phone versus physical tracing outcomes. The study results will inform national and county-level approaches for scaling up APS to prioritize the critical factors for success. The elicitation and documentation of IPV in the study also provides insights into the safety of APS. In addition, those identified as probable or confirmed IPV cases through the study are referred to gender-based violence centers of excellence for appropriate care, following best practices.

The limitations of this study include that some key outcomes, including linkage to care and ART, are self-reported by participants. Furthermore, the proportion of index clients who were adolescents was low (11%). To overcome these challenges, we are assessing viral loads that are at 12-months post APS to objectively assess ART linkage and adherence. In addition, our study did not enroll clients who were men who have sex with men, who are a key population that should be targeted via APS; future work is warranted in this area. Finally, some of the findings regarding implementation fidelity, integration, acceptability, demand, and cost may not be generalizable to other settings outside Kenya.

Scaling up APS is an important step in providing HTS in sub-Saharan countries as it facilitates testing of undiagnosed individuals in populations such as men who have been challenging to reach with the existing HTS. We are conducting this APS scale-up study using implementation science methodologies and existing HTS infrastructure in Homa Bay and Kisumu counties. We expect to complete a 12-month follow-up for participants by May 2021 and will share results with NASCOP as well as in local and international practice-oriented and scientific meetings.

In response to the COVID-19 pandemic and accompanying challenges in conducting standard APS [31], we are shifting to investigate the effectiveness of self-testing for testing partners identified through APS. HIV self-testing is an essential adaptation to the current COVID-19 pandemic to limit the effect of physical distancing measures and lockdowns, particularly on facility- and community-based partner tracing and testing. In addition, the World Health Organization recommends self-testing as an approach to increase access to HTS [32], and it has the potential to both reach partners who would not otherwise participate in standard APS and reduce the costs associated with provider-delivered HIV testing in standard APS. The results of our studies will be used to bridge the gap between clinical research findings and everyday practice and provide guidance on optimal strategies for APS integration into HIV service delivery.

## Appendix

Multimedia Appendix 1 Intimate partner violence screening questionnaire.

## Abbreviations

APS: assisted partner services
ART: antiretroviral therapy
DREAMS: Determined, Resilient, Empowered, AIDS-free, Mentored, and Safe
HTS: HIV testing services
IDI: in-depth interview
IPV: intimate partner violence
MOH: Ministry of Health
NASCOP: National AIDS and Sexually Transmitted Infection Control Programme
PLWH: people living with HIV
SSA: sub-Saharan Africa

## References

[1] Kharsany AB, Karim QA (2016). HIV infection and AIDS in sub-Saharan Africa: current status, challenges and opportunities. Open AIDS J.

[2] (2020). Fact Sheet - latest global and regional statistics on the status of the AIDS epidemic. UNAIDS.

[3] Sharma M, Ying R, Tarr G, Barnabas R (2015). Systematic review and meta-analysis of community and facility-based HIV testing to address linkage to care gaps in sub-Saharan Africa. Nature.

[4] Musheke M, Ntalasha H, Gari S, McKenzie O, Bond V, Martin-Hilber A, Merten S (2013). A systematic review of qualitative findings on factors enabling and deterring uptake of HIV testing in sub-Saharan Africa. BMC Public Health.

[5] Suthar AB, Ford N, Bachanas PJ, Wong VJ, Rajan JS, Saltzman AK, Ajose O, Fakoya AO, Granich RM, Negussie EK, Baggaley RC (2013). Towards universal voluntary HIV testing and counselling: a systematic review and meta-analysis of community-based approaches. PLoS Med.

[6] Dalal S (2017). Improving HIV test uptake and case finding with assisted partner notification services. AIDS.

[7] Katz DA, Wong VJ, Medley AM, Johnson CC, Cherutich PK, Green KE, Huong P, Baggaley RC (2019). The power of partners: positively engaging networks of people with HIV in testing, treatment and prevention. J Int AIDS Soc.

[8] (2016). Guidelines on HIV self-testing and partner notification. World Health Organization.

[9] Rutstein SE, Brown LB, Biddle AK, Wheeler SB, Kamanga G, Mmodzi P, Nyirenda N, Mofolo I, Rosenberg NE, Hoffman IF, Miller WC (2014). Cost-effectiveness of provider-based HIV partner notification in urban Malawi. Health Policy Plan.

[10] Cherutich P, Golden MR, Wamuti B, Richardson BA, Ásbjörnsdóttir KH, Otieno FA, Ng'ang'a A, Mutiti PM, Macharia P, Sambai B, Dunbar M, Bukusi D, Farquhar C (2017). Assisted partner services for HIV in Kenya: a cluster randomised controlled trial. Lancet HIV.

[11] Brown LB, Miller WC, Kamanga G, Nyirenda N, Mmodzi P, Pettifor A, Dominik RC, Kaufman JS, Mapanje C, Martinson F, Cohen MS, Hoffman IF (2011). HIV partner notification is effective and feasible in sub-Saharan Africa: opportunities for HIV treatment and prevention. J Acquir Immune Defic Syndr.

[12] Mahachi N, Muchedzi A, Tafuma TA, Mawora P, Kariuki L, Semo B, Bateganya MH, Nyagura T, Ncube G, Merrigan MB, Chabikuli ON, Mpofu M (2019). Sustained high case‐finding through index testing and partner notification services: experiences from three provinces in Zimbabwe. J Intern AIDS Soc.

[13] Tih PM, Chimoun FT, Mboh Khan E, Nshom E, Nambu W, Shields R, Wamuti BM, Golden MR, Welty T (2019). Assisted HIV partner notification services in resource-limited settings: experiences and achievements from Cameroon. J Int AIDS Soc.

[14] TEMPRANO ANRS 12136 Study Group (2015). A trial of early antiretrovirals and isoniazid preventive therapy in Africa. N Engl J Med.

[15] Myers RS, Feldacker C, Cesár F, Paredes Z, Augusto G, Muluana C, Citao S, Mboa-Ferrao C, Karajeanes E, Golden MR (2016). Acceptability and effectiveness of assisted human immunodeficiency virus partner services in Mozambique: results from a pilot program in a public, urban clinic. Sex Transm Dis.

[16] Sharma M (2018). Assisted partner notification services are cost-effective for decreasing HIV burden in western Kenya. AIDS.

[17] Masyuko SJ, Cherutich PK, Contesse MG, Maingi PM, Wamuti BM, Macharia PM, Bukusi DE, Otieno FA, Spiegel HM, Dunbar MD, Golden MR, Richardson BA, Farquhar C (2019). Index participant characteristics and HIV assisted partner services efficacy in Kenya: results of a cluster randomized trial. J Int AIDS Soc.

[18] Mills EJ, Beyrer C, Birungi J, Dybul MR (2012). Engaging men in prevention and care for HIV/AIDS in Africa. PLoS Med.

[19] Sharma M, Barnabas RV, Celum C (2017). Community-based strategies to strengthen men's engagement in the HIV care cascade in sub-Saharan Africa. PLoS Med.

[20] Glasgow RE, Lichtenstein E, Marcus AC (2003). Why don't we see more translation of health promotion research to practice? Rethinking the efficacy-to-effectiveness transition. Am J Public Health.

[21] UNAIDS Kenya AIDS indicator survey dataset 2012. UNAIDS.

[22] Curran GM (2012). Effectiveness-implementation hybrid designs: combining elements of clinical effectiveness and implementation research to enhance public health impact. Medical care.

[23] (2020). KENPHIA 2018 preliminary report. National AIDS and STI Control Programme (NASCOP).

[24] National AIDS and STI Control Programme (2018). A guidance document for the delivery of HIV Self-Testing and assisted partner notification services in Kenya. Ministry of Health, Kenya.

[25] Hartung C, Lerer A, Anokwa Y, Tseng C, Brunette W, Borriello G (2010). Open data kit: tools to build information services for developing regions. Proceedings of the 4th ACM/IEEE International Conference on Information and Communication Technologies and Development.

[26] Carroll C, Patterson M, Wood S, Booth A, Rick J, Balain S (2007). A conceptual framework for implementation fidelity. Implement Sci.

[27] Grépin KA, Reich MR (2008). Conceptualizing integration: a framework for analysis applied to neglected tropical disease control partnerships. PLoS Negl Trop Dis.

[28] Berman P, Pallas S, Smith AL, Curry L, Bradley EH (2011). Improving the delivery of health services : a guide to choosing strategies. Health, Nutrition, and Population (HNP) Discussion Paper.

[29] Guest G, MacQueen KM (2008). Handbook for Team-Based Qualitative Research.

[30] Grosfeld-Nir A, Ronen B, Kozlovsky N (2007). The Pareto managerial principle: when does it apply?. Int J Prod Res.

[31] Lagat H, Sharma M, Kariithi E, Otieno G, Katz D, Masyuko S, Mugambi M, Wamuti B, Weiner B, Farquhar C (2020). Impact of the COVID-19 Pandemic on HIV Testing and Assisted Partner Notification Services, Western Kenya. AIDS Behav.

[32] (2019). Consolidated guidelines on HIV testing services for a changing epidemic. World Health Organization.

