# Supplementary material for: Using Assisted Partner Services for HIV Testing and the Treatment of Males and Their Female Sexual Partners: Protocol for an Implementation Science Study
Source: JMIR Res Protoc. 2021 May 20;10(5):e27262. doi: 10.2196/27262 (PMC8176338; doi:10.2196/27262)
Supplement: Multimedia Appendix 1 [file resprot_v10i5e27262_app1.docx]

**APPENDICES**

*Appendix 1. IPV Screening Questionnaire*

| **ASSISTED PARTNER NOTIFICATION INTERVENTION SCREENING FORM**  ***Partners: those who came in for testing after being notified of HIV exposure by health advisor* | |
| --- | --- |
| **SECTION I. INTIMATE PARTNER VIOLENCE (IPV) QUESTIONS**  “*I would like to ask you some questions about your current and past relationships. We want to make sure you are safe.”* | |
| 1. Have you ever been in a relationship with a person who has physically hurt you?  No **(GO TO Q2)**  Yes | 1.1 If yes, how long ago were you physically hurt by someone you were in a relationship with (the most recent time)?  More than 6 months ago  In the past 6 months  1.1a If in past 6 months:  In the past 1 month  Not in the past 1 month |
| 2. Have you been in a relationship with a person who threatens, frightens, insults, or treats you ***badly***?  No **(GO TO Q3)**  Yes | 2.1 If yes, how long ago were you theatened or frightened by someone you were in a relationship with (the most recent time)?  More than 6 months ago  In the past 6 months  2.1a If in past 6 months:  In the past 1 month  Not in the past 1 month |
| 3. Have you been in a relationship with a perosn who forces you to participate in sexual activities that make you feel uncomfortable?  No **(GO TO Q4)**  Yes | 3.1 If yes, how long ago were you forced to particiate in sexual activities that made you feel uncomfortable (the most recent time)?  More than 6 months ago  In the past 6 months  3.1a If in past 6 months:  In the past 1 month  Not in the past 1 month |
| 4. Do you think any of these things could happen to you if you decide to receive assisted partner notification services? | No  Yes |
| 5. Are you pregnant? *(Ask only if female)* | No  Yes |
| **SECTION II. IPV RISK CATEGORY** | |
| 6. Which IPV risk category is the client in?  *(Refer to Q1, Q2 and Q3. Choose the first category, top to bottom, with matching criteria.)* | HIGH  *(any ticked “In the past 1 month” in Q1,1, 2.1, 3.1)*  MODERATE  *(any ticked “yes” in Q1, Q2, or Q3 but none ticked “in the past 1 month” in Q1.1, 2.1, 3.1)*  LOW  *(all ticked “No” in Q1, 2, 3)* |
| **SECTION III. ELIGIBILITY** | |
| 7. Is the client eligible to receive assisted partner notification services?  *(Tell the subject if their eligible. If not eligible, tell them why.)* | No  *(if any of the following are true; check all that apply)*  <15 years minor and no parental consent  High IPV risk  Yes |
| 8. HTS Counsellor only: Is the client eligible for the study? | No  *(if any of the following are true; check all that apply)*  <15 years minor  High IPV risk  Pregnant *(if female)*  Currently in HIV care or treatment *(if Index   Client)*  Yes |
| **SECTION IV. IPV MONITORING PLAN** **ALL High or Moderate IPV Risk Subjects, regardless of aPS eligibility | |
| 9. Was client referred? | No (**Go to Q10)**  Yes **(Go to Q11)** |
| 10. Why was client not referred? *(Tick one)* | Not in a relationship  Refused  Other  *Specify:*__________________________________  (End Form) |
| 11. Where was client referred to? | __________________________________________ |
| **SECTION V. IPV MONITORING FOLLOW-UP**  **ONLY APS-Eligible, Moderate IPV Risk Subjects who are Referred | |
| 12. Client prefers follow up at:  *(Tick one)* | BEGIN - IPV Monitoring Log  while answering remaining questions  Phone: ______________________  Home  Clinic  Other: _______________________ |
| 13. Next follow-up contact scheduled for: | DD / MM / YYYY  *(date must be within next 10 days)* |
